# Unthought-of Problems Regarding Hepatitis D Virus Infection

**Published:** 2010-06-01

**Authors:** Seyed Moayed Alavian

**Affiliations:** 1 Baqiyatallah Research Center for Gastroenterology and Liver Disease, Baqiyatallah University of Medical Sciences, Tehran, I.R. Iran

**Keywords:** Occult Hepatitis D, Delta Infection, HBV, HDV, Carrier

## Abstract

In this editorial I will present some unanswered questions about hepatitis D virus (HDV) infection and some further discussion; everybody is asked to send us their comments.

Hepatitis D virus (HDV) was discovered by Rizzetto et al. [1] and after its discovery, the role of HDV infection in the exacerbation of hepatitis B virus (HBV) infection was revealed. Hepatitis D virus infection occurs worldwide, but data about its incidence and prevalence are different in different parts of the world [2]. The epidemiology of HDV infection is similar to that of HBV, with notable exceptions. It is estimated that approximately 5% of hepatitis B surface antigen (HBsAg) carriers are infected with HDV infection worldwide [3]. The infection had been endemic in the Mediterranean basin, the Middle East, and parts of Africa [4][5][6]. However, the rate of HDV infection has decreased in many countries of the world, due to the introduction of HBV vaccination, subsequently decreasing HBV infection and thus the pool of HBsAg carriers who may also be infected with HDV[7]. Socioeconomic improvements, and measures introduced to control the human immunodeficiency virus (HIV), are also responsible for this decrease. Nonetheless, HDV still continues to represent a public health problem in some parts of the world [8][9][10][11][12].

Thirty years after the discovery of HDV [13], there are still some clinical findings that should be clarified and addressed. I would like to mention some of these issues and ask scientists to think more about them, and send us their comments, as letters to the editor.

## First

An observation: A 36-year-old HBsAg positive man was referred to our clinic after the detection of HBV infection in his family one year earlier. He was totally asymptomatic and the first evaluation showed abnormal alanine aminotransferase (ALT; 60 IU/ml), a high viral load for HBV infection (125,000 IU/ml by Amplicor version 2 test), and the antibody to HDV (anti-HDV Ab) and HDV RNA were positive. I recommended liver biopsy and treatment. Unfortunately, he declined to continue with us. After two years, he came back, and our evaluation showed that he had become HDV RNA-negative without any intervention.

A question: Did seroconversion occur without any therapy, in a non-acute case of hepatitis D, or had it been an HDV RNA-negative case in the first place? Was it a diagnostic mistake?

Comments: First of all, I would like to discuss the sensitivity and specificity of the tests. The anti-HDV Ab is detected by using an enzyme-linked immunosorbent assay (ELISA) kit (Radim SpA, Pomezia, Italy) according to the manufacturer’s protocol with > 98% sensitivity and specificity; and for molecular study [14], RNA was extracted from the patient’s serum by using the QIAmp Viral RNA Mini Kit according to the manufacturer’s instructions (Qiagen GmbH, Germany); and for reverse transcription, 10µl of extracted RNA was used in a reaction tube containing reverse transcription buffer (50 mM Tris-HCl[PH8.3], 50 mM KCl, 4mM MgCl2), 100 pmol random primers,12U of RNase Inhibitor ,60 U of Molony-Murine Leukemia virus reverse transcriptase, 1 mM deoxyribonucleoside triphosphate (dNTP) and 4µl of diethyl pyrocarbonate treated water (DEPC). The reaction was incubated at 42C for 30min on an Eppendorf Personal Thermal Cycler (Eppendorf, Germany). cDNA was amplified using sequence specific primers for HDV, according to the published method [15]. For cDNA amplification, in the first round of the polymerase chain reaction (PCR), 5µl of cDNA was added to a PCR tube containing 2.5µl of 10x PCR buffer, 1.5 mM MgCl2, 0.2mM dNTP, 5U Taq DNA polymerase (Roche, Germany) and100 pmol of each external primer 5413, 8276. For the nested PCR reaction, 2µl of the PCR product from the first amplification were added to the second PCR tube containing internal primers 5414, 5415 and the same reagents as in the first round of the PCR. The amplification products were separated on a 2% agarose gel and an expected 405bp PCR product, visualized by ethidium bromide staining, confirmed the presence of HDV RNA in the patient’s serum. Unfortunately, the sensitivity and specificity of molecular tests is not reliable in Iran. Secondly, there are some data regarding mismatching between anti-HDV Ab positivity and HDV RNA findings, in the literature. I think some patients with a history of confronting HDV infection can eliminate the virus, and be mono-infected with HBV infection. The natural course of HDV is not totally known yet [16], but recovery from acute hepatitis D infection is possible [17]. I think the patient’s age when acquiring HDV infection can affect its course and outcomes. Most Iranian patients co-infected with HDV/HBV acquire the infection in childhood [2][8][18]. On the other hand, there are at least three distinct HDV genotypes, and these genotypes can affect the remission rate and the adverse outcomes of the infection. I would like to mention that the predominant genotype in the Iranian population is type I [14]. On the other hand, the HBV genotypes exert a significant influence on remission rate and adverse outcomes too. Genotype C has a lower remission rate and more adverse outcomes than genotype B [19]. The main HBV genotype identified in Iran has been genotype D, and unfortunately we have not been able to find any data regarding the influence of HBV genotype D on the course of HDV infection in the literature.

What do others say?

## Second

An observation: A 25-year-old man had been HBsAg positive for 2 years, with normal ALT (42 IU/ml) and aspartate aminotransferase (AST; 35 IU/ml) levels, negative hepatitis B e antigen (HBeAg), and positive HDV Ab and HDV RNA in the serum. A liver biopsy was performed and revealed grade 2 and stage zero, according to Knodel scoring.

A question: Can we define it as a case of a carrier state of HDV infection?

Comments: We are not really sure what defines “carrier state” in HDV infection. First of all, I would like to pose a question about the normality of ALT in this case. I think the result of 40 or 45 IU/ml may be high and should really not be considered within the normal range [20]. Second, we need to do the measurement of ALT more than once, and at different times, for better interpretation of the results. Third, it is well known that liver biopsy is associated with a significant rate of false negative results in the diagnosis of chronic hepatitis D infection [21]. In addition, the accuracy of the histological assessment of necro-inflammation and fibrosis are dependent on the size of the specimen.

What do others say?

## Third

An observation: A 25-year-old HBsAg positive man was diagnosed after family screening for HBV infection. His mother was the first in the family to be diagnosed (as cirrhotic) the year before. In addition, two of his brothers and one of his sisters were also affected. The results of laboratory tests in this case were: elevated ALT (98 IU/ml), negative HBeAg and anti HDV Ab. The HBV viral load was determined to be 125 IU/ml by the Amplicor, version 2, test. Liver biopsy revealed severe damage to hepatic cells (grade 8 and stage 4, according to Knodel scoring). Interestingly, HDV RNA was shown to be positive.

Questions: There are many questions here. First, is it enough to require evidence of the anti-HDV Ab in the evaluation of chronic hepatitis B-infected patients? Also, can we rule out HDV infection on this basis? Does using HDV RNA in addition to the anti HDV Ab test as the screening test make a difference, in cases that are suspected to have been affected with HBV and HDV infection during their childhood, directly or vertically?

Comments: I would like to coin a new term with reference to HDV infection “Occult HDV”, and ask you to produce more evidence in the field of HDV diagnosis, especially regarding primers and serological evaluation and an improvement in the sensitivity and specificity of tests.

What do others say?
